# Integration of Complexed Caffeic Acid into Poly(Lactic Acid)-Based Biopolymer Blends by Supercritical CO_2_-Assisted Impregnation and Foaming: Processing, Structural and Thermal Characterization

**DOI:** 10.3390/polym17060803

**Published:** 2025-03-18

**Authors:** Patricia Rivera, Alejandra Torres, Miguel Pacheco, Julio Romero, Marina P. Arrieta, Francisco Rodríguez-Mercado, Julio Bruna

**Affiliations:** 1 Packaging Innovation Center (LABEN), Faculty of Technology, Center for the Development of Nanoscience and Nanotechnology (CEDENNA), University of Santiago de Chile (USACH), Santiago 9170201, Chile; patricia.rivera.f@usach.cl (P.R.); miguel.pacheco.a@usach.cl (M.P.); francisco.rodriguez.m@usach.cl (F.R.-M.); julio.bruna@usach.cl (J.B.); 2 Laboratory of Membrane Separation Processes (LabProSeM), Department of Chemical Engineering, Engineering Faculty, University of Santiago de Chile, Santiago 9170201, Chile; julio.romero@usach.cl; 3 Departamento de Ingeniería Química Industrial y del Medio Ambiente, Escuela Técnica Superior de Ingenieros Industriales, Universidad Politécnica de Madrid (ETSII-UPM), Calle José Gutiérrez Abascal 2, 28006 Madrid, Spain; 4 Grupo de Investigación, Polímeros, Caracterización y Aplicaciones (POLCA), 28006 Madrid, Spain

**Keywords:** active supercritical foams, supercritical carbon dioxide, inclusion complex, food packaging

## Abstract

Conventional techniques for incorporating active ingredients into polymeric matrices are accompanied by certain disadvantages, primarily attributable to the inherent characteristics of the active ingredient itself, including its sensitivity to temperature. A potential solution to these challenges lies in the utilization of supercritical carbon dioxide (scCO_2_) for the formation of polymeric foam and the incorporation of active ingredients, in conjunction with the encapsulation of inclusion complexes (ICs), to ensure physical stability and augmented bioactivity. The objective of this study was to assess the impact of IC impregnation and subsequent foam formation on PLA films and PLA/PBAT blends that had been previously impregnated. The study’s methodology encompassed the formation and characterization of ICs with caffeic acid (CA) and β-cyclodextrin (β-CD), along with the thermal, structural, and morphological properties of the resulting materials. Higher incorporation of impregnated IC into the PLA(42)/PBAT(58) blend was observed at 12 MPa pressure and a depressurization rate of 1 MPa/min. The presence of IC, in addition to a lower rate of expansion, contributed to the formation of homogeneous cells with a size range of 4–44 um. On the other hand, the incorporation of IC caused a decrease in the crystallinity of the PLA fraction due to the interaction of the complex with the polymer. This study makes a significant contribution to the advancement of knowledge on the incorporation of compounds encapsulated in β-CD by scCO_2_, as well as to the development of active materials with potential applications in food packaging.

## 1. Introduction

Polymeric foams are porous microstructures and are defined as a two-phase gas-polymer system [1]. Among the polymers used are expanded polystyrene (EPS) and expanded polyethylene (EPE) [2,3,4], as these possess good barrier properties, which help preserve the freshness of perishable products such as fruits, seafood, vegetables, and some cheeses. However, many of these containers are used only once before being discarded. Because they are not biodegradable, they must be recycled or incinerated, which generates a high volume of waste in landfills and causes significant environmental impact [5]. Based on this, some eco-friendly alternatives have been reported, such as starch, cellulose, and poly lactic acid (PLA) foams [6,7,8]. Among the alternatives, PLA is a bio-based, biodegradable, and biocompatible polymer with properties that make it a potential replacement for polystyrene (PS) [9]. In addition, PLA requires 25–55% less energy to produce than petroleum-derived polymers [10]. Although these polymers may have similar properties to those used commercially, they present certain processing difficulties, so the search for strategies such as copolymerization, development of bionanocomposites, and polymer blending has been resorted to, obtaining products like those on the market and with a low environmental impact [11].

Among the main requirements for the foaming process to occur effectively is to melt or plasticize a polymer matrix to allow the diffusion of gases within its structure [12]. In this sense, these materials can be obtained mainly by using chemical blowing agents, such as chlorofluorocarbons (CFCs), for which alternatives have been sought, including the use of supercritical fluids, due to the growing emphasis on environmental protection [13]. A pure component is in a supercritical state if its temperature and pressure are higher than critical values, allowing it to diffuse solids as a gas and dissolve materials as a liquid. These include carbon dioxide (CO_2_) and nitrogen (N_2_). scCO_2_ is characterized by its relatively low critical temperature and pressure, cost-effectiveness, biocompatibility with the human body (non-toxicity), and current classification by the U.S. Food and Drug Administration (FDA) as a safe biomaterial (GRAS) [14]. CO_2_ can be used in many fields, such as compound preparation and oil field development [15,16]. Such conditions make this method more environmentally friendly, in addition to avoiding the formation of residues after foaming [12]. In this way, Chang and co-workers prepared blends of thermoplastic starch (TPS), silane A (SA), and PBAT for the development of foams at pressures of 17 and 23.8 MPa [17]. In another work, Faba et al. developed PLA foams loaded with carvacrol cocrystals by means of supercritical CO_2_, which showed microcellular sizes and a modulated release by the cocrystal incorporation [18].

Food preservation has been widely studied due to its enormous relevance in social and economic development on a global scale. In recent years, several technologies have emerged to increase and improve food preservation, among them active packaging, in which the packaging interacts positively with the product, prolonging its shelf life thanks to the action of an active agent, which can be incorporated into the packaging material or be part of the polymeric packaging. The economic viability and the environmental benefits of active packaging compared to commercial packaging have been discussed in literature [19,20]. Phenolic acids are found in various foods of plant origin. They are formed by the substitution of hydrogen atoms in a benzene ring by a carboxyl group and a hydroxyl group [21]. They stand out for their antioxidant activity due to radical scavenging by hydrogen atom donation. In this context, 3,4-dihydroxycinnamic acid, also known as caffeic acid (CA), is a notable phenolic compound [22]. CA is a prevalent compound in a wide variety of plants, fruits, and propolis samples [23]. It exhibits diverse bioactivities, such as antiviral, antimicrobial, and anti-inflammatory [24,25,26]. In addition to its use as a crosslinker for materials such as chitosan, the study by Pei et al. using PLA films with CA immobilized on chitosan effectively decreased the browning, respiration rate, and microorganism-induced deterioration of Agaricus Bisporus, thus reducing oxidative damage and delaying aging [27]. Meanwhile, Luzi and co-workers, by casting process, developed active films of 5 and 15 wt% poly (vinyl alcohol-co-ethylene) (EVOH), obtaining higher thermal stability due to the addition of CA [23].

Recent studies have suggested that the use of cyclodextrins (CDs) can enhance the functionalities of the active, such as increasing the antioxidant activity of an active compound. This is achieved by including the active in its truncated conical form, which has a hydrophilic surface and a hydrophobic cavity [28]. CDs are cyclic oligosaccharides of glucose units linked by an α-(1,4)-glucosidic linkage [29]. In addition, its use as a molecular inclusion technique has been shown to improve chemical stability and provide controlled release, which could allow the development of packaging with continuous and prolonged diffusion, as well as reduce or avoid certain preservatives [30,31,32]. On the other hand, an increase in the bioactivity of actives has also been found, which could be crucial for the development of active packaging in which the active does not meet the required antioxidant power or in which the active compound incorporated into the material is low [33,34,35].

Based on this, this work aims to encapsulate CA in a β-cyclodextrin by molecular inclusion, as well as to evaluate the effect of the incorporation of inclusion complexes (β-cyclodextrin/caffeic acid) in PLA and PLA/PBAT mixtures by serial impregnation and supercritical foaming. The study focuses on evaluating the effect of the structure of the developed materials on the structural and morphological thermal properties.

## 2. Materials and Methods

### 2.1. Materials

Two commercial PLA/PBAT blends (BASF, Ludwigshafen, Germany), one composed of 42% PLA and 58% PBAT (trade name: Ecovio^®^ F2224) [36] and the other with a composition of 4% PLA, 84% PBAT, and 12% inert particles/additives (trade name: Ecovio^®^ F23B1) [37], were supplied by Entec Polymers, Chile (Santiago, Chile). Poly (lactic acid) (PLA) 2003D (specific gravity 1.24; MFR g/10 min) was purchased from Natureworks^®^ Co (Minnetonka, MN, USA). Carbon dioxide (99.9% purity) was obtained from Linde (Santiago, Chile). Caffeic acid (CA) (≥98% HPLC) and 2,2-diphenyl-1-picrylhydrazyl (DPPH) were obtained from Sigma-Aldrich (Madrid, Spain). β- cyclodextrin (≥98%) was obtained from Merck (Darmstadt, Germany). Chromatographic-grade acetonitrile, absolute ethanol, and other analytical-grade reagents from Merck S.A. (Darmstadt, Germany).

### 2.2. Formation of Inclusion Complexes (IC) Based on β-CD/CA

The IC was prepared according to the coprecipitation method previously reported with some modifications [38]. For this, (5 g/0.044 mol) of β-CD was added slowly to 50 mL of ethanol/water aqueous solution in a 1:2 ratio while this was kept stirring at 50 °C for 2.5 h and 270 rpm on a magnetic stirrer with a thermocouple attached. Pure caffeic acid (0.7936 g/0.044 mol) was then added in a 1:1 molar ratio in an amber flask to protect it from possible degradation by light. The mixture was stirred with a magnetic stirrer for 30 min at 50 °C, then it was cooled to room temperature for 1 h and after that time it was refrigerated for 24 h to favor the precipitation of the inclusion complexes. The sample was then filtered using filter paper and washed numerous times with 10% (*v*/*v*) ethanol to remove any possible free CA from the precipitate. Finally, the recovered solid phase was dried in a CoolSafe 55-4 Pro freeze dryer (Labogene, Lillerød, Denmark). The inclusion complex was stored in hermetic amber vials at room temperature until further characterization.

### 2.3. PLA and PLA/PBAT Films Preparation by Extrusion Process

The plastic films were obtained by subjecting various polymer resins to a drying process at 60 °C for 24 h and a melt-extrusion process using a Scientific Labtech^®^ LTE20 twin-screw extruder (Praksa, Muang, Samutprakarn, Thailand) located at the Packaging Innovation Center (LABEN-Chile) of the University of Santiago de Chile. Extruder temperature profiles were maintained at 185–195 °C [39] for PLA films and 170−195 °C for PLA/PBAT blends [40]. The screw speed was set at 42 rpm, and a chill roll was used at 0.9 rpm. Films with thicknesses between 500 and 600 µm were obtained and later cut into 4 × 1.5 cm pieces, whose mass and thickness were measured and then stored in a desiccator.

### 2.4. Supercritical Impregnation of Inclusion Complex

**Figure 1:**
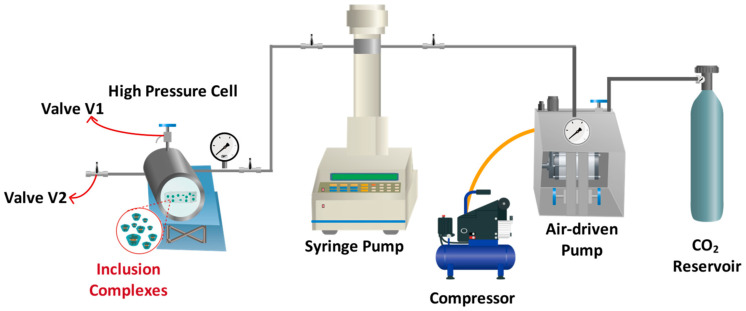
Outline of the experimental setup used for sequential supercritical impregnation and foaming processes.

The initial phase of material processing, encompassing impregnation experiments, was conducted at the Membrane Separation Processes Laboratory (LabProSem) within the Department of Chemical Engineering and Bioprocessing at the University of Santiago de Chile. PLA and PLA/PBAT films developed by the process described in Section 2.3 were processed by supercritical impregnation to incorporate the inclusion complex. The impregnation process (Figure 1) was conducted within a high-pressure cell having a volume of 100 mL and maintained at a constant temperature of 40 °C. The thermostatic resistance surrounding the cell ensured the temperature was consistently regulated throughout the process [39]. CO_2_ was loaded into the system employing an ISCO 500D syringe pump (Lincoln, NE, USA), operated at a constant pressure regime during each impregnation cycle.

The impregnation experiments were carried out by loading a quantity of IC equivalent to 10 mg of caffeic acid (337 mg IC) in a high-pressure cell to ensure the saturation condition of the impregnation phase, as used for films containing only pure CA, as reported in previous work [41], since these were considered the best operating conditions obtained for pure CA impregnations. For this, five samples of material with a surface area of 6 cm^2^ each, with a thickness between 500 and 600 µm, were placed in the high-pressure cell, with a vial containing the amount of IC mentioned above. The supercritical impregnation series were carried out at pressures of 12 or 15 MPa at a constant temperature of 40 °C for 3 h to reach equilibrium conditions. After this time, the system was depressurized at 0.1 or 1 MPa/min with a micrometer valve (V1) to characterize and quantify the amount of impregnated active. The operating conditions are described in Table 1. It should be noted that, for each impregnation condition, the tests were performed in duplicate.

### 2.5. Supercritical Foaming of PLA and Blends

As delineated in Section 2.4, the impregnated materials underwent a supercritical foaming process following impregnation. The foams derived from PLA and PLA/PBAT blends were produced from materials that had previously undergone impregnation in the presence of a co-solvent. As illustrated in Figure 1, the foaming process was conducted. The samples were placed within the high-pressure cell, and CO_2_ was introduced into the system, which was maintained at a constant pressure during the supercritical foaming process. The temperature within the high-pressure cell was regulated by a thermocouple attached to the cell. The supercritical foaming process was conducted at temperatures of 130 °C and pressures ranging from 15 to 25 MPa. The samples were maintained under these conditions for a duration of approximately 25 to 30 min. Subsequently, the CO_2_ was expeditiously released at a depressurization rate of 60 MPa/min [39,42] via valve V2 and the samples were stabilized by an air-conditioned cooling system. The foams produced in these experiments were subsequently stored in a desiccator until the stage of characterization. These experiments were performed in duplicate for each condition.

### 2.6. Encapsulation Efficiency

The amount of caffeic acid trapped in the inclusion complex was determined according to the method proposed by Muñoz et al. [38], with some modifications. The active compound was extracted from 2 mg IC with ethanol (20 mL) in hermetically sealed vials using an incubator shaker IST-4075 (Jeio Tech, Daejeon, Republic of Korea) at 150 rpm and 25 °C for 24 h. The insoluble phase (β-cyclodextrin) was separated by centrifugation, and the supernatant was analyzed spectrophotometrically with UV-1601 Rayleigh (BFRL, Beijing, China) at a wavelength of 242 nm.

The compound was quantified using a previously established calibration curve with different solutions of caffeic acid in ethanol with concentrations ranging from 0 to 30 ppm (R^2^ = 0.99). From this analysis, the efficiency (EE) was calculated using Equation (1):

(1) $$\mathrm{EE}\;[\%]=\frac{\mathrm{Release}\;\mathrm{CA}\;\mathrm{content}}{\mathrm{Theorical}\;\mathrm{CA}\;\mathrm{content}}*\;100$$

### 2.7. Quantification of Active Agent in Impregnated Films

To determine the amount of caffeic acid present in the films and foams, the Folin-Ciocalteu method reported by [43] was used with some modifications. A sample of film or foam was cut into small pieces and placed in a 25 mL amber volumetric flask to protect it from light and possible degradation. A mixture of 1 mL of Folin-Ciocalteu reagent and distilled water was then added and shaken for 2 min. Then 4 mL of sodium carbonate at 2% *w*/*w* was added, and the solution was made up with distilled water. The samples were measured by UV-visible spectrophotometry (UV 1601, Rayleigh) at 760 nm. The time was determined by the time taken for the samples to reach equilibrium concentration. The content of total phenols was determined by means of a calibration curve with the concentration of caffeic acid. For this purpose, a stock solution of 200 mg/L was prepared and diluted to concentrations in the range of 0.08–1.8 mg/L. The measurements were performed in duplicate and expressed as mg of caffeic acid per mg of pure material (film or foam).

### 2.8. Characterization of Inclusion Complexes and Materials Obtained (Films and Foams)

#### 2.8.1. Nuclear Magnetic Resonance (NMR)

^1^H NMR spectra were recorded for β-cyclodextrin and the inclusion complex dissolved in D_2_O using a Varian Mercury 400 MHz spectrometer. Chemical shifts were measured relative to the peak at 4.80 ppm, due to the solvent (D_2_O) [44]. The ^1^H NMR chemical shifts for the inclusion complex and β-cyclodextrin were determined. The difference in chemical shift value was considered by Equation (2), where δ_free_ is the chemical shift of the pure component and δ_complex_ is the chemical shift of the complex [45].

(2) $$∆\delta =\delta_{\mathrm{complex}}-\delta_{\mathrm{free}}$$

#### 2.8.2. Attenuated Total Reflectance Fourier Transforms Infrared (ATR-FTIR) Spectroscopy

The FTIR spectra of β-CD, CA, and IC were performed using potassium bromide (KBr) pellets, while the materials obtained (films and foams) were obtained by direct contact of a piece of material. An ALPHA spectrometer Platinum (Bruker, Billerica, MA, USA) equipped with an attenuated total reflection (ATR) diamond crystal accessory was employed for this purpose. The spectrometer employed the OPUS v7 software suite, which was configured to perform 64 scans per specimen within a wavelength range spanning from 400 to 4000 cm^−1^.

#### 2.8.3. Morphological Properties

The morphologies of the different foam samples were analyzed by scanning electron microscopy (SEM) using a Jeol JSM-5410 scanning microscope (Jeol, Shanghai, China) with accelerating voltage at 20 kV. Cell size was measured using ImageJ v1.54g software and was obtained by measuring the maximum diameter of each cell. To determine the cell size distribution, the size of at least 75 cells in the central part of the cross section of the cryofractured foam sample was considered.

The bulk density (kg/m^3^) of the pre-foamed (ρ_p_) and post-foamed (ρ_f_) samples was determined using a pycnometer by the water displacement method according to ASTM D792-20 [46,47]. Cell densities (NC) were calculated using Equation (3).

(3) $$\mathrm{NC}=\frac{\left(1-\frac{\rho_{f}}{\rho_{p}}\right)}{{(10}^{-4}*d^{3})}$$

On the other hand, the expansion coefficient (ER) of the foamed samples was obtained using Equation (4) [42]:

(4) $$\mathrm{ER}=\frac{\rho_{p}}{\rho_{f}}$$

#### 2.8.4. Thermogravimetric Analysis (TGA)

To determine the thermal stability of the inclusion complex, as well as to evaluate the effect of supercritical processing (impregnation or foaming) on the structural characteristics of the polymeric mixture. Thermogravimetric analysis (TGA) was used to determine the type of degradation and also to evaluate the thermal stability of the different films and foams developed in this work. This analysis was performed at 10 °C/min, between 25 and 700 °C, under nitrogen atmosphere, in an SDT 2960 DSC-TGA (TA Instruments, New Castle, DE, USA).

#### 2.8.5. Differential Scanning Calorimetry (DSC)

The crystallization behavior of pure polymers, films, and foams of PLA/PBAT blends was performed using a DSC model 822e (Mettler Toledo, Columbus, OH, USA) differential scanning calorimeter (DSC). Samples (5–7 mg) were heated from −50 °C to 200 °C at a rate of 10 °C/min in a nitrogen atmosphere [48]. The degree of crystallinity of the films and foams was calculated by the following Equation (5):

(5) $$x_{c}\left(\%\right)=\frac{{∆H}_{m}-{∆H}_{cc}}{w\cdot {∆H}_{m0}}\times 100\%$$

where ∆H_m_ is melting enthalpy, ∆H_cc_ is the cold-crystallization enthalpy, w is the weight fraction of PLA in the blend, and ∆H_m0_ is the melting enthalpy in 100% crystalline PLA, which has a value of 93.6 J/g [49].

### 2.9. Statistical Analysis

An analysis of variance (ANOVA) and Fisher’s test were applied to evaluate the mean differences (with a confidence interval of 95 %) of the results of the thermal properties (DSC and TGA), using the statistical program Statgraphics Plus 5.1 (StatPoint^®^, Inc., Warrenton, VA, USA).

## 3. Results and Discussions

### 3.1. Characterization of Inclusion Complex IC

It is known that the ability of the CD to form inclusion complexes is strongly influenced by the size, shape, hydrophobicity, and form of the host molecule [26]. In addition, there are thermodynamic interactions between the different components of the system (cyclodextrin, host, and solvent), as there must be a favorable energetic driving force that attracts the host to the CD. The inclusion complexes formed by β-cyclodextrin (β-CD) and caffeic acid (CA) used in this study were characterized by an entrapment efficiency of 25%, similar to that reported by Kalogeropoulos and co-workers [50], who used β-CD to encapsulate propolis extract containing caffeic acid among its constituents. Efficiency was measured for each constituent of the extract using an ethanol solution, where 19.4% efficiency was obtained. These results could be related to the fact that a pH equal to 5 in the ethanol-water solution causes the CA structure to be deprotonated in the carboxylic acid group in its COO^-^ form, which causes an increase in the polarity of the active ingredient [51,52]. On the other hand, the presence of ethanol can lead to a decrease in the polarity of the medium, which weakens the driving force for inclusion complex formation [53].

**Figure 2:**
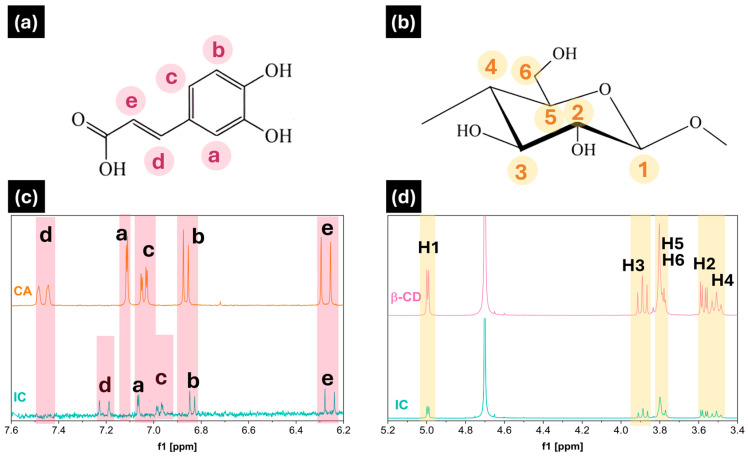
Structures of (**a**) caffeic acid and (**b**) β-Cyclodextrin. Partial ^1^H-NMR partial spectra (**c**) caffeic acid (CA) and β-Cyclodextrin/caffeic acid (IC); (**d**) β-Cyclodextrin (β-CD) and β-Cyclodextrin/caffeic acid (IC), in D_2_O.

The molecular inclusion of CA in the hydrophobic cavity of the β-CD was evidenced by one-dimensional proton nuclear magnetic resonance (^1^H-NMR), where the bands associated with the hydrogens belonging to the host and the guest were analyzed. Figure 2 shows the chemical structures of both compounds (CA and β-CD) and the partial ^1^H-NMR of CA, β-CD, and IC in D_2_O. The chemical shifts (δ) of the internal (H-3 and H-5) and external (H-1, H-2, H-4, and H-6) protons of the β-cyclodextrin and inclusion complex [54], CA (H-a, H-b, H-c, H-d, and H-e), and inclusion complex are shown in Table 2.

In Figure 2c, with respect to the CA and IC spectra, there is a shift mainly of the hydrogens associated with the aromatic ring of caffeic acid (H-b, H-c, and H-d), which could interact with the hydrophobic cavity of the cyclodextrin, while the most polar groups of the molecule, -COOH and hydroxyl groups, would be outside the more hydrophilic cavity. This result has been reported by Chao and coworkers [51], who studied the molecular inclusion of hydroxypropyl cyclodextrin with caffeic acid. On the other hand, Figure 2d would confirm the interaction of the aromatic ring due to the displacement of the internal protons (H-3 and H-5) of the β-CD.

As shown in Table 2, the highest Δδ values can be observed at H-b, H-c, and H-d for CA/IC and H-5 with respect to β CD/IC. As reported by other authors [38,55,56,57], the difference in chemical shifts confirms the formation of the inclusion complex, as they indicate significant variations between the cyclodextrin cavity and the guest (CA). These findings were corroborated by FTIR and TGA analysis.

**Figure 3:**
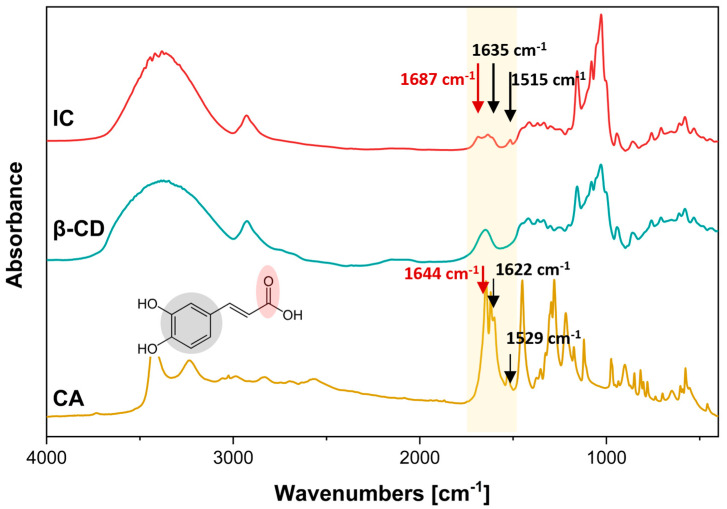
FTIR spectra of different compounds: CA; β-CD; IC.

FTIR analyses allowed the identification of characteristic bands of the inclusion complex through vibrational and rotational motions of the molecular bonds. The formation of the IC inclusion complex involves the interaction of hydrogen bonds due to hydrophobic interactions of the internal cavity of the β-CD. The generated interactions are represented by the increase, decrease, or shift of certain characteristic frequencies in the spectrum. Figure 3 shows the spectra of CA, β-CD, and IC.

**Figure 4:**
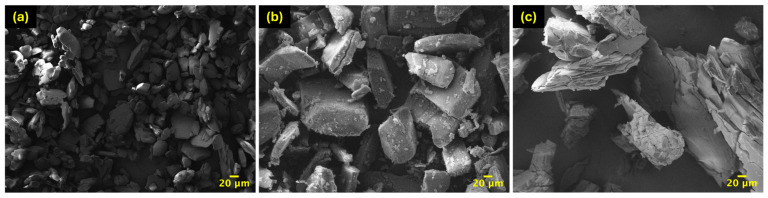
SEM micrographs of (**a**) β -CD; (**b**) IC at 500× and (**c**) CA at 500×.

In the spectrum of Figure 3, it is illustrated that β-CD exhibited a broad band at 3386 cm^−1^, indicative of OH-stretching vibrations, and a band at 2926 cm^−1^, associated with the C-C bond tension of the polysaccharide. Additionally, a band was observed at 1649 cm^−1^. The band at 1251 cm^−1^ is attributed to the asymmetric stretching vibration of the glycosidic bond, while the intense absorption at 1029 cm^−1^ is due to the C-O-C stretching of the alcohol [58]. The latter is also evidenced by the band at 3386 cm^−1^, which is related to the OH-stretching vibrations. In the case of CA, the OH groups of its aromatic ring can be observed at 3433 cm⁻^1^ [55]. The IC spectrum (Figure 4) shows a shift and decrease in the CA bands in the carbonyl groups (C=O) from 1644 cm^−1^ to 1687 cm^−1^ and in the aromatic moiety (C=C) at 1622 cm^−1^ to 1635 cm^−1^ and at 1515 cm^−1^. Finally, these results allow us to suggest that the CA is within the hydrophobic cavity of the cyclodextrin [57].

The morphological structure of β -CD, CA, and IC particles was characterized by SEM. As shown in Figure 4, the surface of the IC is rough and exhibits cracks in accordance with that inclusion complex synthesized by Zhang and coworkers [59]. However, the structural features of the host cyclodextrin were preserved when the IC. In contrast, both β-CD and CA particles displayed a smooth surface with square-shaped, consistent with micrographs reported in the literature [60,61].

**Figure 5:**
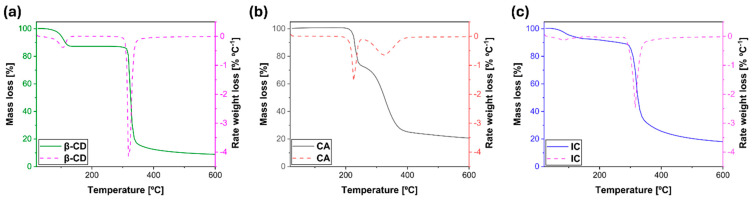
TG and DTG thermograms of (**a**) β-cyclodextrin (β-CD), (**b**) caffeic acid (CA), and (**c**) inclusion complex (IC) samples.

Finally, with regard to thermal stability, Figure 5 presents the thermograms (TG and DTG) of IC and the pure compounds (CA and β-CD). The β-CD exhibited a mass loss at two distinct temperatures, below 100 °C, attributable to water loss, and subsequently at around 330 °C, corresponding to the thermal decomposition of the β-CD [62]. CA, on the other hand, exhibited stability above 150 °C and underwent a two-step thermal decomposition process. The initial step, characterized by a weight loss of 20%, involved the melting and degradation of CA at 228 °C. The subsequent step, attributed to acid decarboxylation, occurred at 335 °C and resulted in a weight loss of approximately 60% [23,63].

On the other hand, in Figure 5c, the IC shows a first region corresponding to a slight loss of mass of 6% below 100 °C, which is attributed to the presence of water [64]. In addition, a second region around 315 °C shows the highest mass loss. As for the endothermic peaks (150 and 228 °C) associated with the decomposition of CA, none are observed. This would indicate that the formation of IC protects CA from thermal degradation.

### 3.2. Quantification of Caffeic Acid (CA) in Impregnated Films and Foams

**Figure 6:**
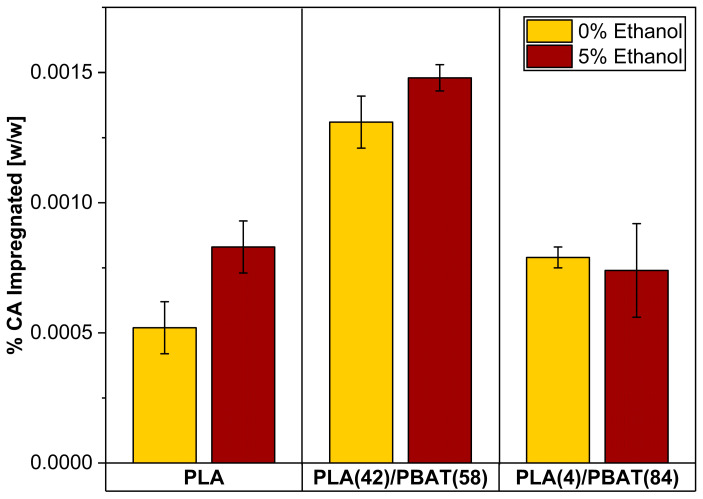
Amount of caffeic acid (expressed as % *w*/*w*), incorporated in PLA, PLA(42)/PBAT(58) and PLA(4)/PBAT(84) films, by supercritical impregnation of the inclusion complex.

With regard to the sensitivity of the method, a detection and quantification limit equivalent to 3.32 µg/L and 10.05 µg/L was obtained, respectively. The lowest concentration value of caffeic acid quantified was 12.6 µg/L. The IC impregnation process in PLA and PLA/PBAT blends contemplated the best operating conditions for samples incorporating pure CA, which were obtained from a previous work [41] that impregnated the same polymeric matrices. Based on this, Figure 6 presents the results obtained, considering a constant temperature of 40 °C, with a CO_2_ density of 718 (12 MPa) and 780 kg/m^3^ (15 MPa) [65], as well as a cosolvent concentration of 0 and 5% *w*/*w* ethanol.

The incorporation of ethanol as a cosolvent (5% *w*/*w*) into the polymer matrix resulted in an augmentation of CA, both for pure CA and IC impregnations. The addition of small amounts of scCO_2_-soluble polar cosolvent (ethanol) has been demonstrated to enhance the plasticizing effect of CO_2_ on the polymer. Furthermore, the incorporation of ethanol into the supercritical phase enhances the solubility of the active compound by improving the polarity of the high-pressure phase and, consequently, its solvating capacity. This was also reported by Bitencourt et al. [66], who analyzed the solubility of caffeic acid in scCO_2_ at different pressures and temperatures and obtained a significant increase when ethanol was used as a cosolvent, since the presence of a polar solvent induces specific interactions, such as hydrogen bonding interactions, between the solute and the solvent.

With respect to the PLA(4)/PBAT(84) blend, a different trend was obtained compared to the other polymeric matrices when the impregnation was performed in the cosolvent presence. This discrepancy can be attributed to CO_2_ solubility in PBAT, which is 8.5 g CO_2_/100 g polymer (15 MPa and 40 °C), as compared to that of PLA, which is 22.6 g CO_2_/100 g polymer (15 MPa and 40 °C) [67]. Additionally, the presence of CaCO_3_ in the blend may also influence the impregnation of the active compound. The efficiency of the supercritical impregnation process depends largely on the interactions in a ternary system composed of the active agent, the polymeric matrix, and CO_2_.

Regarding the low impregnation rates obtained when using IC, it can be explained by the low solubility of the active compound in scCO_2_ due to the high polarity of CA and β-CD (due to hydroxyl groups oriented in the outer layer of the molecule), hindering the transport of the active towards the polymeric matrix, which leads to low impregnation yields. The low affinity of these compounds in scCO_2_ could explain the low amount of impregnated CA. When comparing these results with those obtained by, which used PLA, PLA(42)/PBAT(58), and PLA(4)/PBAT(84) with and without ethanol under similar operating conditions, with an experimental matrix that only contemplated the use of caffeic acid without cyclodextrins, obtained an impregnation rate of 0.0055% (mg CA/mg polymer) in the PLA(42)/PBAT(58) polymer at a pressure of 12 MPa with a depressurization rate of 1 MPa/min and with the use of ethanol as cosolvent at 5%. Cejudo and coworkers impregnated caffeic acid in polyethylene terephthalate films with polypropylene (PET/PP) using pressures between 10 and 40 MPa and obtained a maximum impregnation rate of 0.006%, which was due to the low solubility of the active in scCO_2_ and the limited affinity of caffeic acid in the films [68]. This accounts for the fact that supercritical impregnation is highly dependent on the interaction and equilibrium of the ternary system, i.e., polymer matrix/active agent/scCO_2_. This includes the effect of pressure, temperature, and/or structural change of the polymer on the solubility, diffusivity, and partition coefficient in the system [69,70].

Finally, the effect of the supercritical foaming process at 15 and 25 MPa on the impregnated films was evaluated at the conditions of higher incorporation of caffeic acid with and without IC, which is presented in Table 3.

Table 3 shows a decrease in the amount of CA in all polymers after foaming. This is due to the fact that the impregnated foams are again exposed to scCO_2_, so that the inclusion complex is resolubilized by the fluid, dragging it at the moment of generating the depressurization of the system [71]. As for the differences in the amounts of compound loaded in the polymer, it can be attributed to the pressures of 15 and 25 MPa used in the foaming process, which causes the density of scCO_2_ to vary at 262 and 471 g/mL, respectively. As expected, the higher the pressure, the higher the density of scCO_2_ and thus the ability of both compounds to dissolve in the medium [72,73]. Although this variable affects the compounds equally, CA still has a higher affinity for the medium compared to IC, because it has a non-polar end, so it solubilizes better in the medium. On the other hand, when depressurizing the system, less compound is lost when using IC. This occurs because the CA is inside the cavity of the β-CD, which hinders the interaction of the molecule with the medium. In addition, the cyclodextrin presents in its outer layer a large amount of OH groups, a phenomenon that can be observed in the FTIR spectrum of IC. This polarity difference causes a low affinity between IC and scCO_2_, reducing the amount of compound entrained during depressurization [66].

### 3.3. Characterization of the Materials Obtained (Films and Foams)

#### 3.3.1. ATR-FTIR Analysis

The structural analysis of the films and foams (denoted with the subscript F) developed was conducted using Fourier transform infrared spectroscopy—attenuated total reflectance (FTIR-ATR) analyses. These analyses were performed to ascertain the characteristic functional groups of each polymer. These groups were identified by distinct bands that indicate vibrations and stretching associated with their chemical bonds.

**Figure 7:**
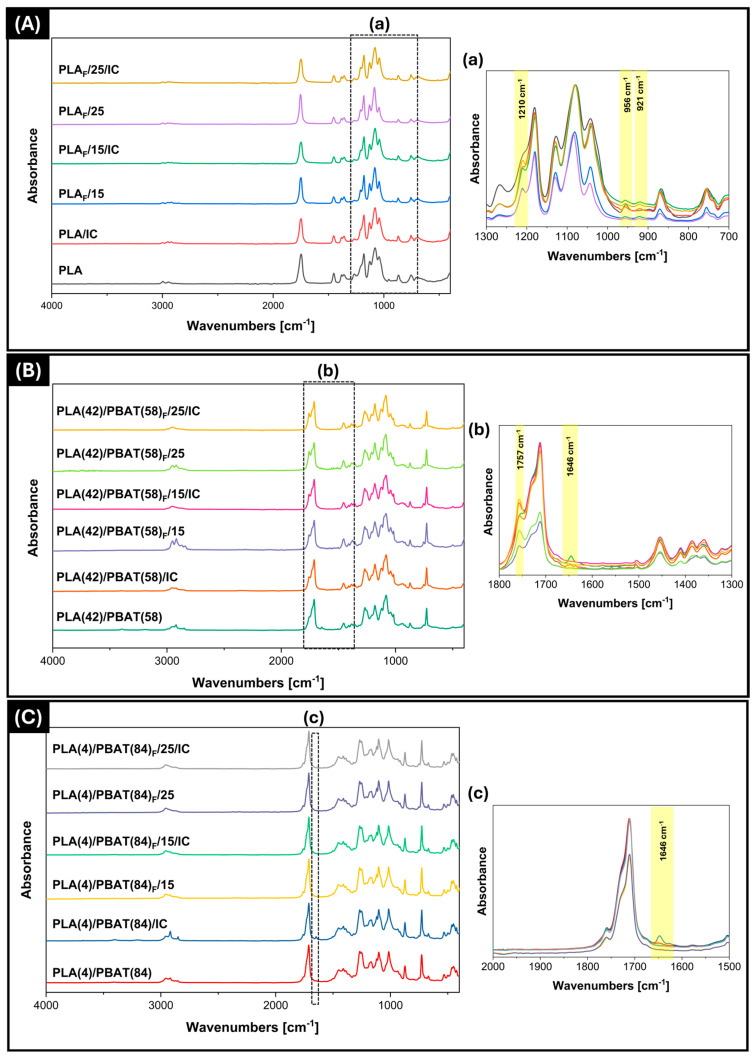
FTIR spectra of films and foams (F as subindex) of: (**A**) PLA, (**B**) PLA(42)/PBAT(58), (**C**) PLA(4)/PBAT(84), with and without the inclusion complex. (**a**) PLA spectrum in the region 1300–700 cm^−1^, (**b**) PLA(42)/PBAT(58) spectrum in the region 1800–1300 cm^−1^, (**c**) PLA(4)/PBAT(84) spectrum in the region 2000–1500 cm^−1^.

The characteristic bands of PLA, such as carbonyl groups (C=O) at 1747 cm^−1^, stretching of methyl groups (CH_3_) at 1453 and 1380 cm^−1^ [74,75], and two signals associated with asymmetric C-O-C and C-O vibrations at 1180 and 1079 cm^−1^ [76], respectively, could be observed in all samples shown in Figure 7A. In addition, the bands observed at 867 cm^−1^ and 754 cm^−1^ are attributed to C-C bond stretching, a characteristic feature of the amorphous and crystalline phases of PLA [39,77].

In contrast, a distinguishing feature of PLA relative to PBAT is the presence of phenyl rings, as evidenced by the spectra in Figure 7B,C at a wavenumber of 1015 cm^−1^ [48,54]. Furthermore, the analysis revealed the presence of signals from the C=O stretching of the carbonyl and ester groups in both mixtures at 1712 cm^−1^, a band at 1267 cm^−1^ belonging to the stretching of the C-O ester groups, and a strong signal at 727 cm^−1^ corresponding to the stretching of the CH_2_ groups [78].

While PLA (Figure 7A(a)) films exhibited clear variations at 1210 cm^−1^ due to the vibration of the C-O-C group resulting from the asymmetric oscillation of CH_3_, additional peaks emerged at 956 and 920 cm^−1^. These peaks corresponded to relative changes in the PLA amorphous fraction (decreased due to the presence of IC) and the α-crystalline phase, respectively.

The increase in bands may be due to the effect of IC on PLA crystallization as demonstrated in the DSC tests below, as well as the rearrangement of amorphous to crystalline phases, which may be in individual regions or throughout the polymer. Meaurio and coworker [79] evaluated the effect of PLLA crystallinity by Fourier infrared spectroscopy, showing that as the crystallinity of the polymers increases, the intensity of the bands also increases.

Finally, changes in the band at 1646 cm^−1^ were observed in the films with and without IC of PLA(42)/PBAT(58) and PLA(4)/PBAT(84) (Figure 7B(b),C(c)). These changes may be related to the CO_2_ impregnation process, as CO_2_ produces a swelling effect that generates polymer chain mobility and subsequently reduces IR penetration. This may indicate that the swelling effect of the polymer matrix is partially irreversible and/or the polymer chain order is modified after depressurizing the system [80].

#### 3.3.2. Thermogravimetric Analysis (TGA)

To assess the effect of the incorporation of the inclusion complex, the supercritical impregnation process, and the pressure variation during foaming on the thermal stability of PLA films and PLA/PBAT blends, thermogravimetric analyses were performed. The values obtained from the thermograms are shown below in Table 4, in bold italics, the results for pure and impregnated films are shown, while the foams are shown with a letter F as a subindex.

Regarding the pure materials (prior to the supercritical impregnation process), PLA presents a one-stage thermal degradation, with an initial degradation temperature (T onset) equal to 344 °C. On the other hand, the PLA(42)/PBAT(58) mixture presented a 2-stage thermal degradation, the first (337 °C) corresponds to the presence of PLA, and the second to PBAT (388 °C), which has a higher thermal stability with respect to PLA because it has a benzene ring in its structure that allows it to withstand higher temperatures.

The PLA(4)PBAT(84) mixture exhibited a third degradation stage at 586 °C, indicative of the presence of inorganic matter. According to previous research by other authors, this corresponds to the decomposition of calcium carbonate (CaCO_3_) into carbon dioxide and calcium oxide (CaO) [81]. The observed reduction in the thermal stability of PLA is attributable to the catalytic effect of CaCO₃ in depolymerizing the ester bond, a process that is facilitated by the presence of a metal ion (Ca^2^⁺) and the subsequent formation of free radicals and reactive terminal groups during the decomposition of polyesters [82,83].

On the other hand, in the samples impregnated with the inclusion complex, changes were only observed in the PLA(42)/PBAT(58)/IC samples, this would agree with the results in the quantification analyses, since this sample presented a greater amount of impregnated active agent. The increase in the initial degradation temperature of PBAT could be related to its chemical structure, since it presents two carbonyl groups, with which the inclusion complex could be interacting.

In the case of foamed samples, it is important to clarify that the foams without active compound were only exposed once to scCO_2_, while the active ones were subjected to supercritical impregnation and then foamed. With respect to the active and pure samples, no statistically significant differences in their thermal stability were observed, suggesting that supercritical reprocessing of the material does not contribute to changes in the different materials.

#### 3.3.3. Differential Scanning Calorimetry (DSC)

**Figure 8:**
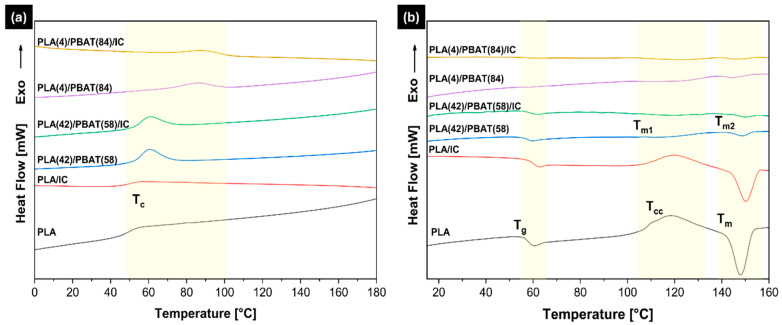
(**a**) Cooling and (**b**) 2nd heating curves in the DSC thermograms of films of PLA, PLA(42)/PBAT(58), PLA(4)/PBAT(84), with and without the inclusion complex.

Differential scanning calorimetry (DSC) was utilized to ascertain the impact of the samples with the most substantial IC incorporation during the impregnation process on their transition temperatures. Figure 8 and Table 5 present the results obtained for PLA and PLA/PBAT blend impregnated films, as well as films that were not exposed to CO_2_ and IC, in order to discern potential structural alterations induced by the impregnation of the active compound.

As demonstrated in Table 5, the PLA/PBAT blends exhibited two glass transition regions, at temperatures of −31 °C for PBAT and 56 °C corresponding to the melting point of PLA. This observation indicates that the blends are immiscible and exhibit a two-phase structure [42], a phenomenon analogous to that reported by Chiu and coworkers [84]. In their study, PLA/PBAT blends with varying proportions were obtained by means of injection molding, with glass transition temperatures for PBAT ranging from −35 to −30 °C and for PLA from 57 to 61°C. In comparison between the PLA(42)/PBAT(58) and PLA(4)/PBAT(84) mixture, the latter presented a statistically significant increase in Tm of PBAT and X_c_ of PLA. The rise in X_c_ PLA can be ascribed to the synergistic impact of two elements: firstly, the elevated percentage of PBAT (84%) within the mixture, and secondly, the presence of calcium carbonate (CaCO₃), which constitutes the remaining 12% of the inert matter stipulated for this blend. The concomitant presence of PBAT and CaCO₃ has been demonstrated to enhance the crystallization behavior of PLA, as evidenced by studies indicating that rigid CaCO₃ particles function as nucleating agents, thereby contributing to enhanced crystallinity [83,85,86].

The thermograms presented in Figure 8 reveal that pure PLA, with and without IC, exhibited an exothermic peak of cold crystallization T_cc_ around 120 °C during the second heating (Figure 8b). This phenomenon is attributed to the recrystallization of PLA. This confirms what was reported by other authors about the slow crystallization rate of PLA, since it does not occur in a single step during cooling, which generates a partial realignment of its molecular chains during the second heating.

On the other hand, the thermal behavior of the PLA/PBAT blend films was different. During the cooling step, the DSC thermograms of both blends (Figure 8a) showed an exothermic signal, mainly related to the induction of PLA crystallization by the presence of PBAT. Tc values ranged between 60 and 85 °C for the PLA(42)/PBAT(58) and PLA(4)/PBAT(84) blends, respectively. The increase in temperature and the decrease in the intensity of the exothermic peak for the mixture presenting a higher proportion of PBAT could be due to the influence of CaCO_3_ which, acting as a nucleating agent, also contributes to accelerating the crystallization rate. Therefore, in both PLA/PBAT blends, recrystallization of PLA molecular chains did not occur during the second heating, leading to the disappearance of the T_cc_ peak [87].

With respect to IC incorporation, an increase in the melting temperature of PLA and PBAT was observed, which is related to the increase in Van der Wals forces. The increase in Van der Waals forces can be attributed to the interaction of the polymer chains with IC. On the other hand, the PLA(42)/PBAT(58) blend showed a decrease in the enthalpy of fusion for the PBAT fraction, which is attributed to the decrease in the crystalline zones [88]. However, no statistically significant changes were observed for X_c_, which could be related to the distribution of inclusion complexes in the polymer matrix.

Taking into account that crystallization affects cell growth, the relationship between foam cell morphology and the crystals it contains was evaluated. For the purpose of analysis, the initial heating of the DSC thermograms was examined to illustrate the alterations induced by the incorporation of PBAT into the mixtures and the subsequent processing with scCO_2_. The results of T_m_, H_m_, and X_c_ of PLA for foams with and without IC are presented in Table 6.

Table 6 shows that both the control samples and the samples with IC presented differences mainly in the enthalpy of fusion (ΔH_m_) and the degree of crystallinity (%X_c_). According to the literature, this phenomenon is attributed to the crystallization that occurs during the foaming process [49]. The absorption of additional CO_2_, which is a consequence of heightened pressure, leads to substantial swelling of the polymer matrix. This, in turn, results in a plasticizer effect that facilitates the reorientation of polymer chains at a reduced free energy [89]. While the foams previously impregnated with IC presented a statistically significant increase in X_c_, only in PLA(42)/PBAT(58) at 15 MPa, which presented a higher amount of impregnated active. This could be related to the heterogeneous nucleation effect caused by the incorporation of the inclusion complex into the polymeric matrix, as reported by other authors [88,90].

### 3.4. Scanning Electron Microscopy (SEM)

Given that the morphology of foams is characterized by size (d), shape, cell density (NC), expansion coefficient (ER), and bulk density (ρ_f_), the different foam samples obtained were analyzed by scanning electron microscopy (SEM). The results of this analysis are presented in Table 7.

**Figure 9:**
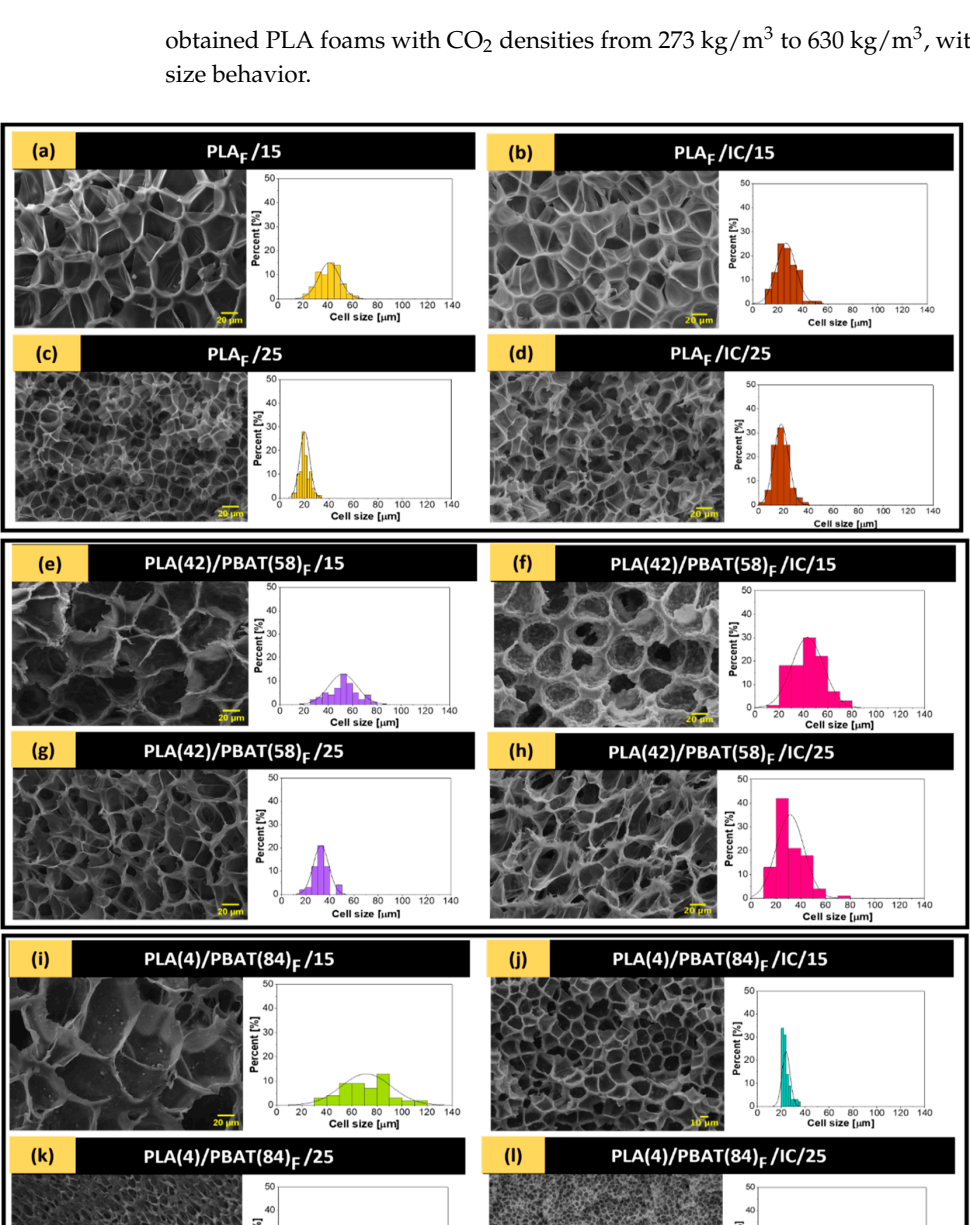
Left side: SEM micrographs of the cross section of 500× cold fractured samples of IC impregnated polymers (**a**) PLA_F_/15, (**b**) PLA_F_/IC/15, (**c**) PLA_F_/25, (**d**) PLA_F_/IC/25, (**e**) PLA (42)/PBAT(58)_F_/15, (**f**) PLA (42)/PBAT(58)_F_/IC/15, (**g**) PLA (42)/PBAT(58)_F_/25, (**h**) PLA (42)/PBAT(58)_F_/IC/25, (**i**) PLA(4)/PBAT(84)_F_/15, (**j**) PLA(4)/PBAT(84)_F_/IC/15, (**k**) PLA(4)/PBAT(84)_F_/25, (**l**) PLA(4)/PBAT(84)_F_/IC/25.

From Table 7, an increase in cell density can be observed, due to the presence of PBAT causing a heterogeneous nucleation effect. Figure 9 show a decrease in pore size is observed with increasing system pressure, and at the same time, the increase in CO_2_ density (262.03–471.26 kg/m^3^) [65], which leads to a higher dissolution of the polymer in the supercritical phase, as well as a smaller cell size [91]. The influence of amorphous and crystalline structures on the formation of PLA foams is a subject of considerable interest. The presence of PBAT has been shown to modify the cell size, density, and expansion coefficient of PLA/PBAT-based foams, thereby influencing their crystalline behavior, as evidenced by DSC analysis. Results, like those obtained by Milovanovic et al. [92], who obtained PLA foams with CO_2_ densities from 273 kg/m^3^ to 630 kg/m^3^, with similar pore size behavior.

On the other hand, impregnation with IC in the polymers followed the trend of variation in cell diameter and density with increasing pressure, except for the PLA/IC polymer (Figure 9b,d), where at 25 MPa a larger cell diameter and a lower cell density were obtained compared to that formed at 15 MPa. This may be due to the decrease in the crystallinity of this polymer at 25 MPa, where a less crystalline structure impairs cell formation, favoring cell coalescence and therefore forming larger pores [93]. However, this phenomenon is not seen in the PLA(4)/PBAT(84) polymer, where its crystallinity behaves in the same way when varying the pressure. This may be due to the fact that in the mixture of this polymer there is calcium carbonate, which can act as a stabilizer and act on the coalescence of foam cells independently of the crystallinity, which can also impact the drastic growth of cell density when foaming at 25 MPa [94].

Lower expansion rates can be seen when foams with IC are used, which may be due to the fact that the active agent interferes with the crystallinity of the polymers, leading to the formation of homogeneous cells that hinder the expansion rate [95]. Regarding the effect of pressure on the polymers, there is a tendency for the expansion rate to decrease as the pressure increases to 25 MPa with the exception of PLA(42)/PBAT(58) polymer, where the opposite behavior is observed. This phenomenon is related to the changes in crystallinity observed in the DSC analysis. Specially, a higher crystallinity percentage suggests a greater degree of molecular ordering, however, which in turn may hinder scCO_2_ diffusion and bubble formation [96].

## 4. Conclusions

This study presents the characterization of PLA and PLA/PBAT films and foams impregnated with a caffeic acid/β-cyclodextrin inclusion complex, focusing on their thermal, structural, and morphological properties. NMR, FTIR, and TGA analyses confirmed the successful inclusion of caffeic acid in β-cyclodextrin. Additionally, DSC analysis of the PLA and PLA/PBAT samples (both films and foams) further validated the incorporation of the inclusion complex through impregnation with scCO_2_.

The foams previously impregnated with IC presented a lower loss of the active compound during the foaming process, it is related to the polarity of the external layer of the β-CD. This effect could be beneficial for obtaining a material with potential use in food packaging, with antioxidant activity without the need to impregnate a large amount of IC, since according to what has been reported, the molecular inclusion of this type of compound contributes to an increase in antioxidant activity.

Finally, serial impregnation and foaming allowed the production of materials with a variety of cell morphologies. This versatility of the process is explained by several factors related to supercritical CO_2_ desorption and expansion, such as the chemical nature of the active agent, the physicochemical and transport properties of both polymers and their different mechanical properties.

## Figures and Tables

**Table 1:** Conditions for supercritical impregnation of the inclusion complex (IC) in the different films.

Sample	Pressure [MPa]	Depressurization Rate [MPa/min]	Cosolvent Concentration [wt%]
PLA/IC	15	1	0; 5
PLA(42)/PBAT (58)/IC	12	1	0; 5
PLA(4)/PBAT(84)/IC	12	0.1	0; 5

**Table 2:** ^1^H-NMR chemical shifts (δ, ppm) for CH protons of pure β-CD, pure CA, and their complexation-induced shifts (Δδ = δ_complex_ − δ_free_).

	δ_Free_	δ_Complex_	Δδ
Ha	7.110	7.150	−0.04
Hb	6.875	6.848	0.027
Hc	7.033	6.940	0.093
Hd	7.445	7.220	0.225
He	6.295	6.367	−0.072
H-1	5.088	5.087	0.001
H-2	3.680	3.678	0.002
H-3	3.978	3.976	0.002
H-4	3.598	3.597	0.001
H-5	3.868	3.860	0.008
H-6	3.892	3.888	0.004

**Table 3:** Variation in impregnated caffeic acid in PLA films and PLA/PBAT blends upon foaming.

Sample	Type	Pressure [MPa]	% CA [*w*/*w*]
**PLA/IC**	**Film**	-	**0.00096 ± 0.0001**
PLA/15/IC	Foam	15	0.00085 ± 0.0001
PLA/25/IC	Foam	25	0.00085 ± 0.0001
**PLA(42)/PBAT(58)/IC**	**Film**	-	**0.0015 ± 0.0001**
PLA(42)/PBAT(58)/15/IC	Foam	15	0.0013 ± 0.0001
PLA(42)/PBAT(58)/25/IC	Foam	25	0.0014 ± 0.0002
**PLA(4)/PBAT(84)/IC**	**Film**	-	**0.00082 ± 0.0001**
PLA(4)/PBAT(84)/15/IC	Foam	15	0.00082 ± 0.0001
PLA(4)/PBAT(84)/25/IC	Foam	25	0.00073 ± 0.0001

Films results are shown in bold type.

**Table 4:** Onset of degradation (T_onset_) and maximum degradation temperatures (T_max_) of films and foams with and without IC.

Sample	Pressure [MPa]	Initial Degradation Temperature [°C]			Maximum Degradation Temperature [°C]		
		1	2	3	1	2	3
**PLA**		**344.9 ± 0.9 ^a,b,c^**	**N.D.**	**N.D.**	**361.8 ± 0.9 ^a,b^**	**N.D.**	**N.D.**
**PLA/IC**		**348.1 ± 1.2 ^a^**	**N.D.**	**N.D.**	**363.2 ± 0.7 ^a^**	**N.D.**	**N.D.**
PLA_F_	15	341.8 ±2.3 ^a,b,c,d^	N.D.	N.D.	360.4 ± 1.3 ^a,b^	N.D.	N.D.
PLA_F_/IC		348.2 ± 0.7 ^a^	N.D.	N.D.	362.9 ± 0.5 ^a^	N.D.	N.D.
PLA_F_	25	346.9 ± 1.7 ^a,b^	N.D.	N.D.	362.6 ± 0.4 ^a^	N.D.	N.D.
PLA_F_/IC		347.5 ± 0.5 ^a,b^	N.D.	N.D.	362.9 ± 0.1 ^a^	N.D.	N.D.
**PLA(42)/PBAT(58)**		**337.3 ± 3.3 ^c,d^**	**388.7 ± 1.1 ^d^**	**N.D.**	**358.1 ± 0.9 ^a,b^**	**400.1 ± 2.9 ^j^**	**N.D.**
**PLA(42)/PBAT(58)/IC**		**340.2 ± 0.1 ^b,c,d^**	**392.2 ± 0.2 ^a^**	**N.D.**	**357.7 ± 1.2 ^a,b^**	**403.3 ± 0.1 ^f^**	**N.D.**
PLA(42)/PBAT(58)_F_	15	337.7 ± 1.2 ^c,d^	387.2 ± 1.9 ^e^	N.D.	357.1 ± 0.2 ^a,b^	399.2 ± 0.2 ^k^	N.D.
PLA(42)/PBAT(58)_F_/IC		336.3 ± 0.1 ^d^	391.1 ± 2.1 ^c^	N.D.	355.5 ± 3.1 ^b^	402.6 ± 0.3 ^h^	N.D.
PLA(42)/PBAT(58)_F_	25	335.3 ± 2.6 ^d^	383.9 ± 5.5 ^f^	N.D.	355.6 ± 2.3 ^a,b^	400.8 ± 1.9 ^l^	N.D.
PLA(42)/PBAT(58)_F_/IC		338.2 ± 1.3 ^c,d^	391.1 ± 0.1 ^b^	N.D.	356.9 ± 3.6 ^a,b^	400.2 ± 3.5 ^i^	N.D.
**PLA(4)/PBAT(84)**		**314.5 ± 0.6 ^e,f^**	**378.6 ± 0.7 ^k^**	**555.5 ± 5.5 ^a^**	**328.9 ± 0.9 ^c^**	**403.5 ± 0.3 ^d^**	**586.5 ± 0.5 ^a^**
**PLA(4)/PBAT(84)/IC**		**314.6 ± 0.3 ^e,f^**	**378.9 ± 0.1 ^j^**	**563.3 ± 9.3 ^a^**	**328.1 ± 0.2 ^c,d^**	**404.6 ± 0.5 ^a^**	**597.2 ± 6.8 ^a^**
PLA(4)/PBAT(84)_F_	15	310.2 ± 0.5 ^f^	378.1 ± 3.9 ^l^	552.9 ± 11.7 ^a^	322.0 ± 2.1 ^d^	402.8 ± 1.7 ^g^	592.7 ± 13.3 ^a^
PLA(4)/PBAT(84)_F_/IC		310.6 ± 1.5 ^f^	379.2 ± 0.1 ^h^	559.1 ± 4.9 ^a^	327.2 ± 1.2 ^c,d^	403.9 ± 0.1 ^b^	592.2 ± 3.0 ^a^
PLA(4)/PBAT(84)_F_	25	320.3 ± 5.2 ^e^	384.7 ± 5.3 ^g^	559.6 ± 7.1 ^a^	326.6 ± 3.5 ^c,d^	403.3 ± 1.0 ^e^	597.8 ± 6.3 ^a^
PLA(4)/PBAT(84)_F_/IC		315.3 ± 1.8 ^e,f^	379.1 ± 0.1 ^i^	562.2 ± 6.9 ^a^	329.3 ± 1.3 ^c^	403.7 ± 0.4 ^c^	590.7 ± 1.4 ^a^

N.D. not detected. Films results are shown in bold type. Lowercase letters a–l indicate significant differences between the values of each thermal parameter.

**Table 5:** Thermal parameters of control and impregnated films with and without IC.

Sample	T_g_ PBAT [°C]	T_g_ PLA [°C]	T_m_ PBAT [°C]	H_m_ PBAT [J/g]	T_m_ PLA [°C]	H_m_ PLA [J/g]	X_c_ PLA [%]
PLA	N.D.	58.1 ± 0,2 ^a^	N.D.	N.D.	148.2 ± 0.2 ^b^	29.2 ± 0.4 ^a^	4.1 ± 0.2 ^b^
PLA(42)/PBAT(58)	−31.3 ± 0.1 ^c^	57.4 ± 1.1 ^a^	115.1 ± 0.1 ^d^	7.1 ± 0.4 ^a^	148.9 ± 0.2 ^b^	2.2 ± 0.1 ^b^	5.7 ± 0.2 ^b^
PLA(4)/PBAT(84)	−32.0 ± 2.6 ^d^	56.6 ± 0.3 ^a^	123.4 ± 0.1 ^b^	4.4 ± 0.2 ^c^	145.8 ± 0.1 ^c^	1.5 ± 0.1 ^b^	39.0 ± 2.6 ^a^
PLA/IC	N.D.	58.5 ± 0.3 ^a^	N.D.	N.D.	149.6 ± 0.8 ^a,b^	28.7 ± 0.5 ^a^	4.0 ± 0.2 ^b^
PLA(42)/PBAT(58)/IC	−29.5 ± 0.3 ^b^	56.9 ± 0.2 ^a^	120.2 ± 0.7 ^c^	3.7 ± 0.4 ^d^	151.1 ± 0.4 ^a^	1.3 ± 0.1 ^b^	3.2 ± 0.1 ^b^
PLA(4)/PBAT(84)/IC	−27.9 ± 0.1 ^a^	56.9 ± 0.7 ^a^	123.8 ± 0.6 ^a^	4.3 ± 0.1 ^b^	145.6 ± 0.1 ^c^	1.4 ± 0.1 ^b^	37.1 ± 2.3 ^a^

Lowercase letters a–d indicate significant differences between the values of each thermal parameter. N.D.: Not detected.

**Table 6:** Thermal parameters of control and impregnated foams with and without IC.

Sample	Pressure [MPa]	T_m_ PLA [°C]	ΔH_m_ [J/g] PLA	%X_c_ PLA
PLA_F_	15	153.5 ± 0.6 ^a,b^	46.1 ± 4.3 ^a^	49.2 ± 4.6 ^b,c^
PLA(42)/PBAT(58)_F_		152.5 ± 0.1 ^a,b,c^	9.9 ± 0.7 ^d^	25.1 ± 1.8 ^g^
PLA(4)/PBAT(84)_F_		150.9 ± 0.2 ^b,c,d^	2.0 ± 0.1 ^e^	52.1 ± 2.3 ^a,b^
PLA_F_	25	148.9 ± 0.4 ^d,e,f^	33.0 ± 0.4 ^b^	35.2 ± 0.4 ^e,f^
PLA(42)/PBAT(58)_F_		147.2 ± 0.5 ^e,f^	15.2 ± 2.3 ^c^	38.7 ± 5.9 ^d,e^
PLA(4)/PBAT(84)_F_		147.3 ± 0.2 ^e,f^	1.0 ± 0.1 ^e^	27.8 ± 2.6 ^f,g^
PLA_F_/IC	15	155.1 ± 0.9 ^a^	43.5 ± 1.2 ^a^	46.5 ± 1.3 ^b,c,d^
PLA(42)/PBAT(58)_F_/IC		153.4 ± 3.1 ^a,b^	17.3 ± 2.3 ^c^	43.9 ± 5.8 ^c,d^
PLA(4)/PBAT(84)_F_/IC		150.8 ± 1.1 ^b,c,d^	2.2 ± 0.1 ^e^	59.8 ± 3.4 ^a^
PLA_F_/IC	25	146.4 ± 1.3 ^f^	32.4 ± 2.7 ^b^	34.6 ± 6.4 ^e,f^
PLA(42)/PBAT(58)_F_/IC		147.2 ± 0.1 ^e,f^	15.6 ± 0.1 ^c^	39.6 ± 0.3 ^d,e^
PLA(4)/PBAT(84)_F_/IC		149.8 ± 3.3 ^c,d,e^	1.1 ± 0.1 ^e^	28.0 ± 1.5 ^f,g^

Lowercase letters a–g indicate significant differences between the values of each thermal parameter.

**Table 7:** Cell diameter (d), foam density (ρ_f_), cell density (NC), and expansion rate (ER).

Sample	Pressure [MPa]	d [μm]	ρ_f_ [kg/m^3^]	NC [×10^11^ Cell/cm^3^]	ER
PLA	15	41.1 ± 8.4	104.7 ± 4.8	1.3	9.1
PLA(42)/PBAT(58)		52.0 ± 11.6	269.6 ± 1.5	0.5	3.3
PLA(4)/PBAT(84)		72.9 ± 19.9	203.6 ± 8.7	0.2	5.0
PLA	25	20.3 ± 4.1	197.5 ± 10.6	9.5	4.8
PLA(42)/PBAT(58)		32.7 ± 6.4	140.1 ± 15.6	2.5	6.4
PLA(4)/PBAT(84)		12.2 ± 2.9	295.5 ± 46.7	39.0	3.4
PLA/IC	15	26.7 ± 7.9	319.5 ± 9.8	3.6	3.3
PLA(42)/PBAT(58)/IC		43.7 ± 7.2	380.5 ± 38.2	0.7	3.3
PLA(4)/PBAT(84)/IC		23.9 ± 3.3	273.0 ± 37.4	5.2	2.6
PLA/IC	25	18.1 ± 5.9	181.1 ± 14.5	14.0	5.7
PLA(42)/PBAT(58)/IC		31.1 ± 6.3	152.4 ± 4.2	2.8	3.5
PLA(4)/PBAT(84)/IC		4.5 ± 1.4	335.9 ± 21.4	689.8	6.6

## Data Availability

The data presented in this study are available upon request from the corresponding authors (due to privacy).
